# Integrative Genomic and Functional Approaches Identify FUOM as a Key Driver and Therapeutic Target in Cervical Cancer

**DOI:** 10.1002/cnr2.70306

**Published:** 2025-08-16

**Authors:** Wenzhi Jiao, Shanshan Liu, Jianwei Shi, Minmin Yu

**Affiliations:** ^1^ Department of Gynecology The Second Hospital of Nanjing, Nanjing University of Chinese Medicine Nanjing Jiangsu China; ^2^ Department of Neurosurgery Xuanwu Hospital, Capital Medical University Beijing China

**Keywords:** cervical cancer, FUOM, immune regulation, Mendelian randomization, single‐cell RNA sequencing

## Abstract

**Background:**

Cervical cancer remains a global public health challenge, particularly in regions with limited access to screening and vaccination. While high‐risk HPV infection is the primary cause, the genetic and molecular mechanisms driving cervical cancer progression are not fully understood.

**Objective:**

This study integrates Mendelian randomization (MR) and single‐cell RNA sequencing (scRNA‐seq) to identify causal eQTL‐related genes and explore their roles in tumorigenesis. Functional experiments were conducted to validate key findings.

**Methods:**

MR analysis identified eQTL‐related genes with significant causal associations with cervical cancer. Functional enrichment and Gene Set Variation Analysis (GSVA) revealed their involvement in key pathways. scRNA‐seq explored cell‐specific expression patterns and immune cell infiltration in the tumor microenvironment (TME). In vitro experiments, including qRT‐PCR, siRNA knockdown, migration, proliferation, and colony formation assays, validated the biological roles of pivotal genes.

**Results:**

A total of 307 eQTL‐related genes were identified, enriched in pathways such as Th17 cell differentiation, TNF, and IL‐17 signaling. scRNA‐seq revealed cell‐specific expression of key genes, including FUOM, which was elevated in cervical cancer cells. FUOM knockdown significantly reduced cell proliferation (by 37%, *p* < 0.001), migration (by 43%, *p* < 0.001), and colony formation (by 62%, *p* < 0.001). Regulatory analysis identified miRNAs as upstream modulators of these genes.

**Conclusion:**

This study identifies FUOM as a novel driver gene in cervical cancer progression and highlights its role in tumorigenesis and immune modulation. These findings provide insights into potential biomarkers and therapeutic targets, offering a foundation for personalized treatment strategies.

**Abbreviations**
eQTL: expression quantitative trait loci
GSEA: Gene Set Enrichment Analysis
GSVA: Gene Set Variation Analysis
HPV: human papillomavirus
IVW: inverse variance weighted
ln(OR): logarithm of odds ratios
MR: Mendelian randomization
scRNA‐seq: single‐cell RNA sequencing
SNP: single nucleotide polymorphism
TME: tumor microenvironment

## 1 Introduction

Cervical cancer is a significant global health issue and remains one of the leading causes of cancer‐related morbidity and mortality among women, particularly in low‐ and middle‐income countries [1, 2]. The disease primarily arises from pathological changes in cervical epithelial cells, often triggered by persistent infection with high‐risk human papillomavirus (HPV) types, especially HPV‐16 and HPV‐18. Integration of the viral DNA into the host genome disrupts normal cellular functions, driving the progression from normal cells to precancerous lesions and eventually invasive cancer [3]. Despite the availability of prophylactic HPV vaccines, which have substantially reduced the incidence of HPV infections, the disease continues to pose a major challenge due to uneven vaccine coverage and the lack of regular screening programs in many regions [4]. These limitations underscore the need for alternative strategies that complement existing prevention and treatment approaches.

Early detection of cervical cancer is critical for improving patient outcomes. When diagnosed at an early stage, the disease can often be effectively treated through surgery, radiotherapy, or chemotherapy, leading to favorable survival rates [5]. However, for advanced‐stage cervical cancer, treatment options are limited, and prognosis remains poor [6]. As a result, identifying reliable molecular markers for early diagnosis and understanding the underlying mechanisms of disease progression are priorities in cervical cancer research. Genetic factors, in particular, play an essential role in driving progress in diagnosis, treatment, and prevention strategies [7]. These insights can help bridge the gap between basic research and clinical applications, leading to personalized interventions that improve patient outcomes.

Mendelian Randomization (MR) has emerged as a valuable tool in genetic epidemiology for investigating causal relationships between modifiable exposures and disease outcomes [8]. By utilizing single nucleotide polymorphisms (SNPs) as instrumental variables, MR mitigates confounding factors and reverse causation biases commonly encountered in traditional observational studies [9]. This approach has proven particularly effective in studying complex diseases such as cardiovascular disease, diabetes, and various types of cancer. In the context of cervical cancer, MR can help identify genetic risk factors and prioritize potential therapeutic targets, contributing to a deeper understanding of the disease's etiology.

Moreover, while MR provides a robust framework for establishing causality between genetic variants and disease phenotypes, its findings often lack direct biological validation [10]. This limitation can be addressed by integrating experimental approaches that confirm the functional relevance of identified targets [11]. Functional assays, such as gene silencing using siRNA or CRISPR, can elucidate the roles of key genes in critical oncogenic processes like proliferation, migration, and immune evasion [12]. These experimental validations not only strengthen computational findings but also pave the way for identifying actionable molecular targets for clinical applications [13].

Meanwhile, advancements in single‐cell RNA sequencing (scRNA‐seq) have transformed our ability to study disease mechanisms at the cellular level [14]. Unlike bulk RNA sequencing, scRNA‐seq captures the heterogeneity of individual cells, enabling the identification of distinct cell populations and their gene expression profiles. This is particularly relevant in cervical cancer, where the tumor microenvironment (TME)—comprising immune, stromal, and tumor cells–plays a pivotal role in disease progression and response to therapy [15]. The use of scRNA‐seq provides insights into the cellular interactions within the TME, uncovering immune evasion mechanisms and potential therapeutic targets.

This study uniquely combines Mendelian randomization with single‐cell RNA sequencing to gain a comprehensive understanding of the molecular drivers of cervical cancer. Unlike previous research that primarily focused on either genetic risk factors or the TME, this approach integrates genetic epidemiology with cellular‐level analyses to identify key expression quantitative trait loci (eQTL)‐related genes and their functional roles within specific cellular contexts. This dual perspective bridges the gap between genetic predisposition and cellular behavior, offering a more holistic understanding of cervical cancer biology and opening new avenues for targeted therapy.

In addition to MR and scRNA‐seq, bioinformatics tools such as Gene Set Variation Analysis (GSVA) and Gene Set Enrichment Analysis (GSEA) play an essential role in this research by identifying dysregulated pathways associated with tumor growth and metastasis [16, 17]. These methods provide functional insights into the biological processes underlying cervical cancer and support the exploration of novel therapeutic targets. Furthermore, constructing transcriptional and microRNA (miRNA) regulatory networks facilitates the study of complex gene expression regulation, offering additional opportunities for identifying actionable biomarkers [18, 19, 20].

In this study, we extend the integration of MR and scRNA‐seq by incorporating functional experiments to validate the biological roles of key genes. Specifically, we focus on FUOM, a gene identified through MR as significantly associated with cervical cancer. By combining computational analyses with qRT‐PCR, siRNA knockdown, and functional assays, we aim to confirm FUOM's involvement in critical oncogenic processes, such as proliferation and migration. This integrative approach bridges the gap between in silico predictions and experimental evidence, offering robust insights into actionable targets for personalized treatment strategies.

## 2 Materials and Methods

**FIGURE 1:**
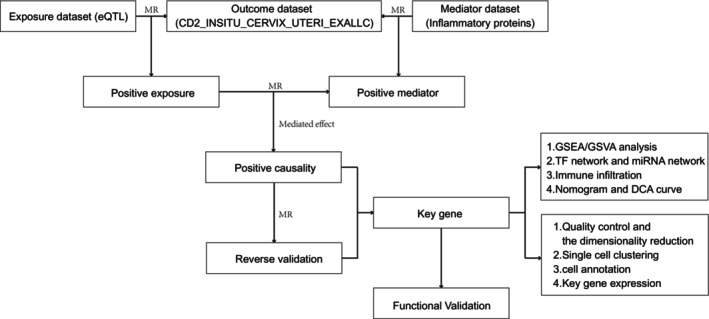
Workflow for Identifying and Validating Key Genes in Cervical Cancer. This figure summarizes the study's workflow, starting with the selection of eQTLs as the exposure dataset and inflammatory proteins as the mediator dataset. Mendelian randomization (MR) was applied to assess causal relationships with cervical cancer outcomes, identify mediators, and evaluate mediated effects. Key genes were subjected to reverse validation and bioinformatics analyses, including pathway enrichment (GSEA/GSVA), regulatory network construction (TF and miRNA), immune infiltration, and clinical prediction using nomograms and decision curve analysis. Single‐cell RNA sequencing further validated key gene expression profiles and their cellular contexts. Finally, functional experiments, including siRNA knockdown and phenotypic assays, confirmed the roles of these genes in tumor progression.

This study was not pre‐registered, as it relied on publicly available genomic datasets and did not involve direct patient recruitment or intervention. The workflow of this study is illustrated in Figure 1, which integrates multiple computational and experimental approaches to identify and validate key genes involved in cervical cancer progression.

Briefly, Mendelian randomization (MR) was employed to investigate the causal relationships between expression quantitative trait loci (eQTL) as the exposure dataset and cervical cancer outcomes. Inflammatory proteins served as the mediator dataset to evaluate mediated effects, and reverse validation was conducted to confirm the robustness of the findings. Identified key genes were subjected to bioinformatics approaches, including pathway enrichment analysis (GSEA/GSVA), transcription factor and miRNA regulatory network construction, immune infiltration analysis, and clinical prediction using nomograms and decision curve analysis (DCA).

To further validate the roles of these key genes, single‐cell RNA sequencing (scRNA‐seq) was used to analyze cell‐type‐specific expression profiles and tumor microenvironment characteristics. Finally, functional validation experiments, including siRNA knockdown, proliferation assays, migration assays, and colony formation assays, were conducted to confirm the biological significance of the key genes in cervical cancer progression.

The following sections describe each methodological step in detail, aligning with the workflow presented in Figure 1.

This figure summarizes the study's workflow, starting with the selection of eQTLs as the exposure dataset and inflammatory proteins as the mediator dataset. Mendelian randomization (MR) was applied to assess causal relationships with cervical cancer outcomes, identify mediators, and evaluate mediated effects. Key genes were subjected to reverse validation and bioinformatics analyses, including pathway enrichment (GSEA/GSVA), regulatory network construction (TF and miRNA), immune infiltration, and clinical prediction using nomograms and decision curve analysis. Single‐cell RNA sequencing further validated key gene expression profiles and their cellular contexts. Finally, functional experiments, including siRNA knockdown and phenotypic assays, confirmed the roles of these genes in tumor progression.

### 2.1 Data Download

Exposure data were obtained from the Expression Quantitative Trait Loci Genotype (eQTLGen) consortium database (https://www.eqtlgen.org). The eQTLGen consortium is dedicated to investigating the genetic architecture of gene expression in blood, providing insights into the genetic basis of complex traits. The current study utilized data from the second phase of the eQTLGen project, which focuses on extensive genome‐wide meta‐analyses in blood samples [21].

Mediator data were sourced from the Genome‐Wide Association Study (GWAS) Catalog, managed by the European Bioinformatics Institute (EBI). The GWAS Catalog is a comprehensive and searchable repository of SNP‐trait associations from published GWAS studies [22]. For this analysis, we used data on 91 inflammation‐related proteins from 14 824 participants of European ancestry. The proteins analyzed included C‐C motif chemokines, fibroblast growth factors, interleukins, and monocyte chemoattractant proteins, among others [23].

Outcome data were obtained from the FinnGen biobank (accession ID: finngen_R11_CD2_INSITU_CERVIX_UTERI_EXALLC), which contains data predominantly from participants of European descent. Cervical cancer cases were defined based on the International Statistical Classification of Diseases (ICD) codes, encompassing 3334 cases and 198 767 controls [24].

RNA sequencing (RNA‐seq) data were downloaded from The Cancer Genome Atlas (TCGA) database (https://portal.gdc.cancer.gov/), the most extensive repository of cancer genomics data, which includes gene expression, microRNA (miRNA) expression, long non‐coding RNA (lncRNA) expression, copy number variation, DNA methylation, and SNP data [25]. For this study, processed cervical squamous cell carcinoma and endocervical adenocarcinoma (CESC) expression data from 198 patient samples (including 3 control samples and 306 tumor samples) were utilized. Additionally, single‐cell RNA‐seq data were obtained from the Gene Expression Omnibus (GEO) database (accession ID: GSE168652), which provided comprehensive single‐cell expression profiles from two samples.

In addition to the aforementioned datasets, miRNA expression data related to cervical cancer were obtained from the HMDD (Human MicroRNA Disease Database) (http://www.cuilab.cn/hmdd). This database includes a collection of 359 miRNAs associated with Uterine Cervical Neoplasms, which was used to identify key miRNAs potentially involved in the regulation of genes identified through the Mendelian Randomization analysis.

### 2.2 Mendelian Randomization Analysis

Mendelian Randomization (MR) analysis was conducted to infer causal relationships between exposures and outcomes. Single nucleotide polymorphisms (SNPs) associated with each exposure at a genome‐wide significance level (*p* < 1e–5) were selected as potential instrumental variables (IVs). Linkage disequilibrium (LD) was assessed, and only SNPs with an *R*‐squared (*R* ^2^) value less than 0.001 (clumping window size = 10 000 kb) were retained. To ensure the strength of the instruments, SNPs with an F‐statistic greater than 10 were included in the analysis, as suggested by previous studies [26]. For the MR analysis, *p* values were primarily interpreted using the IVW method, as it provides the most robust estimates for causal inference under the assumption of no pleiotropy. Causal relationships were evaluated using four statistical methods: Inverse Variance Weighted (IVW), MR Egger, Weighted Median, and Weighted Mode. In cases where the causal relationship involved a single SNP, the Wald ratio method was applied. Leave‐one‐out analysis was performed to assess the robustness of the identified causal relationships [27, 28, 29].

In this two‐sample Mendelian Randomization (MR) study, the genetic variant‐exposure associations were justified based on population homogeneity. Both the exposure data from the eQTLGen consortium and the outcome data from the FinnGen biobank were predominantly derived from individuals of European ancestry, ensuring consistency in genetic architecture between the datasets. Previous studies have shown that such homogeneity reduces potential biases in genetic association studies, thus maintaining the validity of the MR assumptions [30]. Additionally, both datasets followed standardized genotyping protocols with rigorous quality control measures, minimizing technical variability that could otherwise affect the associations between genetic variants and exposures.

There was no overlap of individuals between the exposure and outcome datasets, as the exposure data were obtained from the eQTLGen consortium and the outcome data were sourced independently from the FinnGen biobank. This ensures the two‐sample MR analysis remains free from biases associated with overlapping participants, which could otherwise lead to weak instrument bias and inflated type I error rates [31]. By maintaining independent samples, the reliability of the causal inferences is enhanced.

The validity of the MR assumptions was thoroughly assessed. The relevance assumption was confirmed as all SNPs had F‐statistics greater than 10, ensuring a strong association between the genetic variants and the exposures. The independence assumption was tested using leave‐one‐out sensitivity analysis, which demonstrated that no single SNP disproportionately influenced the overall causal estimates. Finally, the exclusion restriction assumption was evaluated using MR‐Egger regression. The non‐significant MR‐Egger intercept (*p* > 0.05) suggested no evidence of directional pleiotropy. Consistency across the different MR methods, including IVW, MR Egger, and Weighted Median, further supported the robustness of the causal estimates.

These comprehensive assessments confirm that the instrumental variables used in this study meet the core assumptions required for a robust and valid MR analysis, reinforcing the credibility of the causal findings.

### 2.3 Mediation Analysis

Mediation analysis was conducted on exposure‐outcome pairs identified as significant in the MR analysis. The *β* values from the IVW method were used to calculate the mediation effect, following the formula *M* = *β*1 × *β*2. The mediation ratio was determined by dividing the mediation effect by the direct effect (*β*3) of the exposure on the outcome [32, 33].

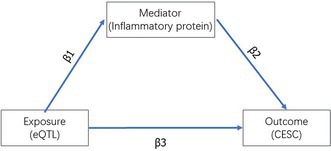

### 2.4 Gene Set Enrichment Analysis

Gene Set Enrichment Analysis (GSEA) was performed to compare signaling pathway activities between patient groups categorized by high and low gene expression levels. Gene sets for the analysis were sourced from the Molecular Signatures Database (MsigDB). Differential pathway expression was evaluated between subtypes, and significantly enriched gene sets (adjusted *p* value < 0.05) were ranked based on their consistency scores. GSEA was employed to explore biological significance and assist in disease subtyping [34].

### 2.5 Gene Set Variation Analysis

Gene Set Variation Analysis (GSVA) was employed to assess transcriptome‐level gene set enrichment in a non‐parametric, unsupervised manner. This method quantifies pathway‐level changes by converting gene‐level expression variations. Gene sets for the analysis were obtained from the Molecular Signatures Database (version 7.0). The GSVA algorithm was used to score these gene sets and to infer potential biological function changes across the samples.

### 2.6 Regulatory Network Analysis of Key Genes

To investigate the regulatory relationships involving key genes, the Cistrome Data Browser (Cistrome DB) was used. Cistrome DB is a comprehensive database for chromatin immunoprecipitation sequencing (ChIP‐seq) and DNase I hypersensitive sites sequencing (DNase‐seq) data. The genome file was set to the human genome version 38 (hg38), and transcription start sites were considered within a 10 kb window. Visualization of the regulatory networks was performed using the Cytoscape software [35].

### 2.7 miRNA Network Construction

MicroRNAs (miRNAs) are small non‐coding RNAs that regulate gene expression by targeting messenger RNA (mRNA) for degradation or translational repression. To explore the miRNA‐mediated regulation of key genes, relevant miRNAs were identified from the HMDD database (359 miRNAs linked to cervical cancer) and the miRNA Database (miRDB). The interactions between miRNAs and their target genes were visualized using Cytoscape [36].

### 2.8 Immune Cell Infiltration Analysis

The composition of immune cells within the tumor microenvironment was estimated using the Cell‐type Identification By Estimating Relative Subsets Of RNA Transcripts (CIBERSORT) method [37]. CIBERSORT utilizes support vector regression to differentiate 22 human immune cell phenotypes, including various T cell, B cell, plasma cell, and myeloid cell subsets, based on the expression of 547 biomarkers. To ensure compatibility with the CIBERSORT algorithm, gene expression data were normalized to transcripts per million (TPM) values, and samples with low RNA integrity or insufficient sequencing depth were excluded during quality control. Statistical significance testing of immune cell proportions was conducted with 1000 permutations to enhance robustness. The relative proportions of these immune‐infiltrating cell types were inferred, and the correlations between immune cell content and gene expression levels were analyzed [38].

### 2.9 Nomogram Model Construction

Nomograms were constructed to visually represent the contribution of various clinical factors and gene expression levels to patient outcomes. These graphical tools were developed based on a multivariable regression model, where each factor's regression coefficient was translated into a score [39]. The total score was then used to predict the probability of 1‐, 3‐, and 5‐year overall survival [40].

### 2.10 Single‐Cell Data Quality Control

Quality control of single‐cell RNA sequencing (scRNA‐seq) data was conducted using the Seurat package. Cells were filtered based on total unique molecular identifier (UMI) count, the number of expressed genes, and the percentage of mitochondrial and ribosomal gene expression. Cells with a high percentage of mitochondrial or ribosomal gene expression and low RNA expression were considered apoptotic and excluded. The median absolute deviation (MAD) method was used to exclude cells that deviated more than 3 MADs from the median [41, 42].

### 2.11 Single‐Cell Data Dimensionality Reduction and Clustering

To ensure data comparability across cells, global normalization was applied using the LogNormalize method, adjusting the total expression per cell to 10 000, followed by log transformation. The CellCycleScoring function was used to calculate cell cycle scores, and highly variable genes were identified using the FindVariableFeatures function. To account for potential batch effects, Harmony was employed after Principal Component Analysis (PCA) for linear dimensionality reduction. Finally, Uniform Manifold Approximation and Projection (UMAP) was applied for nonlinear dimensionality reduction and clustering [43].

### 2.12 Cell Annotation

Cell types were annotated using marker genes identified from the CellMarker and PanglaoDB databases. Automated cell type annotation was performed using the SingleR software. Marker genes for each cell type were identified with the FindAllMarkers function, set to only.pos = TRUE and min.pct = 0.25, ensuring that marker genes were expressed in at least 25% of the cells within each cluster [44].

### 2.13 Statistical Analysis

The validity of the Mendelian randomization (MR) analysis was ensured by adhering to the core assumptions: relevance (instrumental variables [IVs] must be strongly associated with the exposure), independence (IVs must be independent of confounders), and exclusion (IVs affect the outcome solely through the exposure, without pleiotropy). Causal relationships were evaluated using four statistical methods: Inverse Variance Weighted (IVW), MR Egger, Weighted Median, and Weighted Mode. In cases involving a single SNP, the Wald ratio method was used to estimate causal effects.

To ensure the robustness of the instruments, SNPs with an F‐statistic greater than 10 were included in the analysis. Leave‐one‐out analysis was conducted to assess the robustness of the identified causal relationships by excluding one SNP at a time to ensure no single SNP disproportionately influenced the results.

Missing data were handled in accordance with established protocols for large‐scale genomic datasets. As the data were obtained from publicly available resources such as eQTLGen, FinnGen, TCGA, and GEO, most missing values had been pre‐addressed by the original curators. For any remaining missing data, a complete‐case analysis was applied, where observations with missing values were excluded from the analysis to maintain result integrity. Given the robustness of these datasets and stringent quality control during data preprocessing, the impact of missing data on overall results was minimal. Sensitivity analyses were conducted to confirm that excluding incomplete cases did not introduce bias or alter the conclusions.

All statistical analyses were conducted using R software (version 4.3.0). The following R packages were utilized: TwoSampleMR for Mendelian randomization analysis, MRPRESSO for detecting and correcting pleiotropy, GagnonMR for additional MR tools, ieugwasr for accessing GWAS summary data, ggplot2 for data visualization, dplyr for data manipulation, stringr for string processing, forestplot for creating forest plots, and readxl for importing Excel files. Statistical significance was defined as *p* < 0.05, and all tests were two‐sided.

### 2.14 Quantitative Real‐Time PCR (qRT‐PCR)

qRT‐PCR was performed to quantify the expression levels of candidate genes (FUOM, PRKCQ, H2BC21, LAMTOR4) in cervical cancer cells (HeLa) and normal epithelial cells (HaCaT), as well as to confirm the knockdown efficiency of siRNA targeting FUOM.

Total RNA was extracted using TRIzol reagent (Invitrogen, USA) following the manufacturer's instructions. RNA purity and concentration were measured using a NanoDrop spectrophotometer (Thermo Fisher Scientific, USA), and 1 μg of RNA was reverse‐transcribed into cDNA using SweScript All‐in‐One RT SuperMix (Servicebio, G3337). The reaction conditions were 25°C for 5 min, 42°C for 30 min, and 85°C for 5 s.

qRT‐PCR reactions (20 μL) contained 7.5 μL SYBR Green Master Mix (Servicebio, G3326), 0.75 μL each of forward and reverse primers (2.5 μM), 2 μL of cDNA, and 4 μL of nuclease‐free water. Amplifications were performed on a CFX Connect Real‐Time PCR Detection System (Bio‐Rad, USA) with the following cycling conditions: 95°C for 30 s, followed by 40 cycles of 95°C for 15 s and 60°C for 30 s. A melt curve analysis from 65°C to 95°C was performed to confirm amplification specificity. Relative expression levels were calculated using the 2^(−ΔΔCt)^ method, with GAPDH as the internal control. Primer sequences are listed in Table 1.

**TABLE 1:** Primer sequences used for qRT‐PCR.

Gene	Forward primer (5′‐3′)	Reverse primer (5′‐3′)
GAPDH	GGAAGCTTGTCATCAATGGAAATC	TGATGACCCTTTTGGCCTCC
FUOM	GACTTGAAGTTCCCGGCTTC	TTGCCAGGGCTCTCACACAG
LAMTOR4	GCCTGTCGTGGTGTTTGGAA	GACATCAATGGGGTCCCGAC
H2BC21	AACTCCTTGCAACGGACATCTT	TGATTAGGTTGGGTGCGTGA
PRKCQ	CTCAACACGGCTGCTGCTTA	TTTATCCACCTCATCCACGG

### 2.15 Functional Validation

To investigate the functional role of FUOM in cervical cancer, siRNA‐mediated knockdown experiments and functional assays, including cell proliferation, migration, and colony formation assays, were performed using HeLa cells.

#### 2.15.1 Cell Culture and siRNA Transfection

HeLa cells were cultured in Dulbecco's Modified Eagle Medium (DMEM) supplemented with 10% fetal bovine serum (FBS) and 1% penicillin–streptomycin at 37°C in a 5% CO_2_ incubator. siRNAs targeting FUOM (si‐FUOM 1#, si‐FUOM 2#, si‐FUOM 3#) and a non‐targeting control siRNA (si‐NC) were synthesized and transfected into cells using Lipofectamine RNAiMAX (Invitrogen, Thermo Fisher Scientific). Transfection efficiency was validated by qRT‐PCR 48 h post‐transfection.

#### 2.15.2 Cell Proliferation Assay

Cell proliferation was assessed using the MTT assay. HeLa cells transfected with siRNAs were seeded in 96‐well plates at a density of 3 × 10^4^ cells per well. At 0, 24, 48, 72, and 96 h post‐transfection, 20 μL of MTT solution (5 mg/mL) was added to each well and incubated for 4 h. Formazan crystals were dissolved in 150 μL DMSO, and absorbance was measured at 570 nm using a microplate reader (Thermo Fisher Scientific). All assays were performed in triplicate.

#### 2.15.3 Cell Migration Assay

The migratory ability of HeLa cells was evaluated using Transwell chambers with an 8‐μm pore size membrane (Corning). Transfected cells (5 × 10^4^ per well) in serum‐free DMEM were added to the upper chambers, while the lower chambers were filled with DMEM containing 10% FBS. After 24 h at 37°C, cells on the lower membrane surface were fixed with 4% paraformaldehyde, stained with 0.1% crystal violet, and counted under a microscope in five randomly selected fields.

#### 2.15.4 Colony Formation Assay

For the colony formation assay, transfected HeLa cells were seeded in 6‐well plates at a density of 500 cells per well and cultured for 14 days. Colonies were fixed with 4% paraformaldehyde, stained with 0.1% crystal violet, and counted manually. Colonies containing ≥ 50 cells were considered as one colony.

### 2.16 Statistical Analysis

All experiments were performed in triplicate. Results were expressed as mean ± standard deviation (SD). Statistical significance was determined using Student's *t*‐test or one‐way ANOVA in GraphPad Prism 9.0 (GraphPad Software), with a *p* value < 0.05 considered statistically significant.

## 3 Results

### 3.1 Genetic Regulation of Cervical Cancer Risk: A Mendelian Randomization Approach

To explore the genetic basis of cervical cancer susceptibility, we performed a Mendelian randomization (MR) analysis focusing on expression quantitative trait loci (eQTLs). This approach identified genetic variants that influence gene expression and assessed their causal impact on cervical cancer risk. By leveraging comprehensive cervical cancer datasets, we aimed to uncover novel genetic drivers of the disease.

The analysis identified 307 eQTL‐related genes with statistically significant causal effects on cervical cancer risk (IVW *p* value < 0.05). These genes represent key regulatory elements in the genetic architecture of cervical cancer. Notable findings include CD70, FANCD2, PFKP, MORN3, and LEF1, which exhibited strong causal associations and potential relevance in cervical cancer pathogenesis. For instance, PFKP, a key regulator of glycolysis, is implicated in metabolic reprogramming, a hallmark of cancer progression. Similarly, LEF1, a transcription factor in the Wnt signaling pathway, plays critical roles in cell proliferation and differentiation. The identification of MORN3, a less characterized gene, represents a novel finding that warrants further investigation to elucidate its role in cervical cancer.

To ensure the robustness of these findings, sensitivity analyses were conducted using the leave‐one‐out method. These analyses demonstrated that excluding individual single nucleotide polymorphisms (SNPs) did not substantially alter the causal estimates, confirming the stability of the associations. This consistency highlights the reliability of the identified eQTLs as genetic determinants of cervical cancer risk.

These findings provide valuable insights into the genetic underpinnings of cervical cancer and highlight potential therapeutic targets for further investigation. By identifying genes with strong causal relationships, this study lays the groundwork for future research into the molecular mechanisms of cervical cancer and the development of targeted therapeutic strategies.

### 3.2 Mendelian Randomization Analysis of Inflammatory Proteins, eQTLs, and Cervical Cancer

**FIGURE 2:**
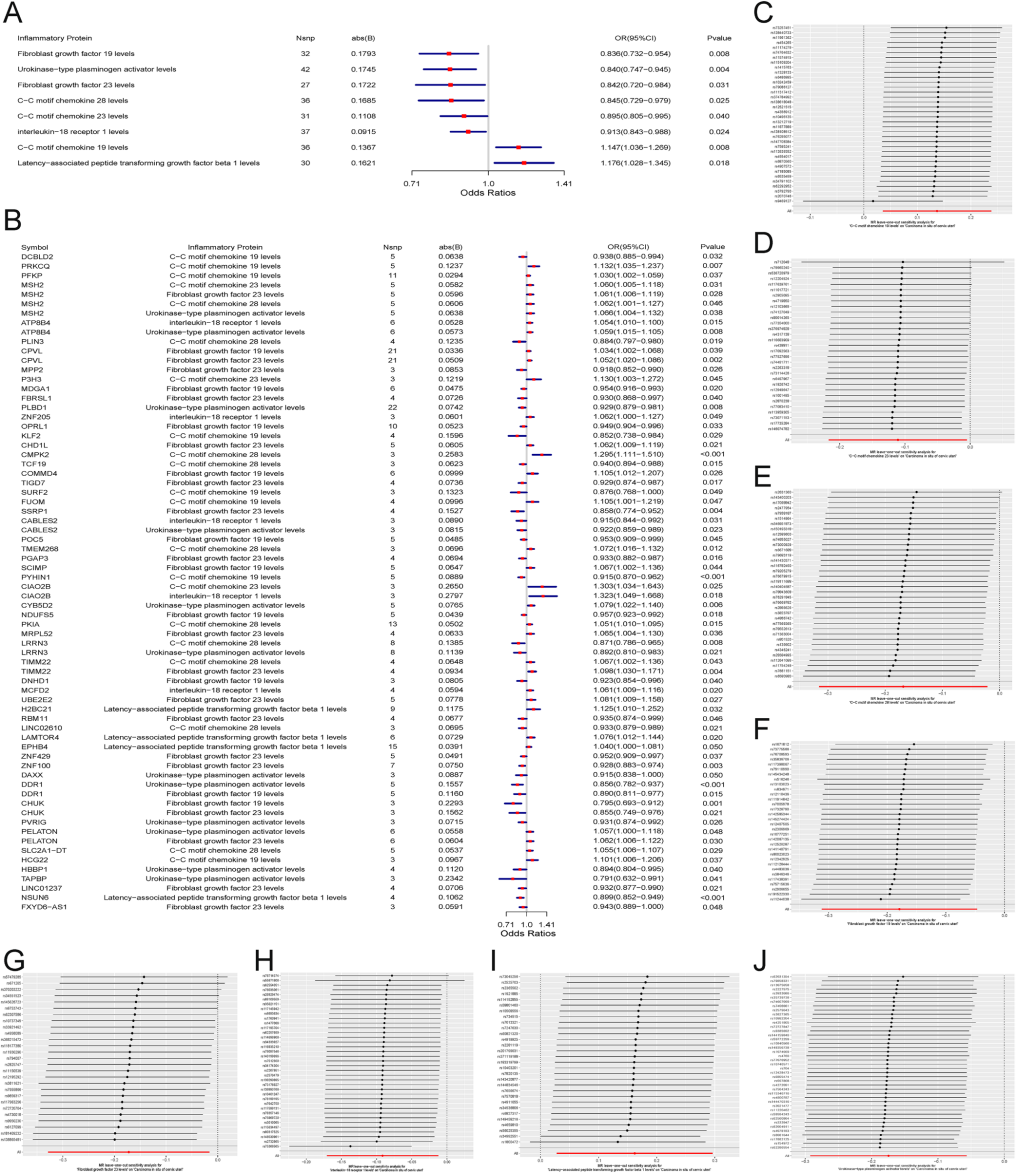
Mendelian randomization and sensitivity analysis linking inflammatory proteins, eQTLs, and cervical cancer risk. (A) Forest plot summarizing the causal effects of eight inflammatory proteins on cervical cancer risk identified through Mendelian randomization (MR). Odds ratios (ORs) with 95% confidence intervals (CIs) are shown as red dots and horizontal lines, respectively. Proteins such as Fibroblast Growth Factor 19 (FGF19), Urokinase‐Type Plasminogen Activator (uPA), Fibroblast Growth Factor 23 (FGF23), C‐C Motif Chemokine 28 (CCL28), and Interleukin‐18 Receptor 1 (IL18R1) were associated with a reduced risk (OR < 1), indicating protective effects. Conversely, C‐C Motif Chemokine 19 (CCL19) and Latency‐Associated Peptide Transforming Growth Factor Beta 1 (LAP‐TGFβ1) were associated with increased risk (OR > 1), suggesting their roles in pro‐inflammatory or immunosuppressive pathways that may promote cervical cancer development. Statistical significance (*p* < 0.05) was observed for all reported associations. (B) Forest plot illustrating the causal relationships between expression quantitative trait loci (eQTLs) and inflammatory proteins. ORs with 95% CIs for gene‐protein pairs reveal both protective and risk effects, providing insights into the genetic regulation of inflammatory pathways linked to cervical cancer. Notable genes include DCBLD2, PRKCQ, PFKP, MSH2, and ATP8B4, which were significantly associated with inflammatory proteins such as CCL19 and LAP‐TGFβ1. (C–I) Leave‐one‐out sensitivity analyses for single nucleotide polymorphisms (SNPs) associated with the eight inflammatory proteins. Each panel corresponds to one protein, showing the impact of excluding individual SNPs on overall causal estimates. Minimal shifts in ORs across these analyses demonstrate the robustness and stability of the identified causal relationships between inflammatory proteins and cervical cancer risk.

To explore the potential causal roles of inflammatory proteins and their genetic regulation in cervical cancer, we performed a comprehensive Mendelian randomization (MR) analysis. This study focused on eight key inflammatory proteins, known for their involvement in both inflammation and cancer biology, and 307 significant eQTLs associated with cervical cancer risk. The results revealed distinct causal relationships, shedding light on the intricate interplay between inflammation, genetic predisposition, and cervical cancer development (Figure 2).

Among the inflammatory proteins analyzed, Fibroblast Growth Factor 19 (FGF19), Urokinase‐Type Plasminogen Activator (uPA), Fibroblast Growth Factor 23 (FGF23), C‐C Motif Chemokine 28 (CCL28), and Interleukin‐18 Receptor 1 (IL18R1) exhibited protective effects against cervical cancer, as evidenced by odds ratios (ORs) less than 1 (Figure 2A). These proteins may mitigate cervical cancer risk through diverse mechanisms, including modulation of immune responses, suppression of inflammation, or inhibition of tumor cell proliferation. For example, FGF19 is known to regulate tissue remodeling and immune homeostasis, which may contribute to its protective role in cancer pathogenesis. In contrast, C‐C Motif Chemokine 19 (CCL19) and Latency‐Associated Peptide Transforming Growth Factor Beta 1 (LAP‐TGFβ1) were associated with an increased risk of cervical cancer (ORs > 1), likely due to their roles in pro‐inflammatory or immunosuppressive pathways. LAP‐TGFβ1, a well‐established immunosuppressive mediator, may promote tumor progression by creating a microenvironment conducive to immune evasion.

Further analysis of the eQTLs regulating these inflammatory proteins revealed 70 significant causal relationships (*p* < 0.05), highlighting the genetic basis of inflammation in cervical cancer. Notable genes such as DCBLD2, PRKCQ, PFKP, MSH2, and ATP8B4 were significantly associated with inflammatory proteins like CCL19 and LAP‐TGFβ1. These findings suggest that these genes influence cervical cancer risk by modulating inflammatory signaling pathways, emphasizing the role of genetic regulation in shaping the inflammatory tumor microenvironment. These insights point to potential molecular mechanisms through which genetic predisposition contributes to cancer development.

To validate the robustness of these findings, we performed sensitivity analyses using the leave‐one‐out method. As shown in Figure 2C–J, the exclusion of individual SNPs did not result in substantial changes in the effect estimates, confirming the stability and reliability of the identified causal relationships. Across all analyses, the odds ratios and confidence intervals remained consistent, further supporting the critical roles of these inflammatory proteins and associated genetic factors in cervical cancer pathogenesis. These results underscore the potential of targeting inflammatory pathways as therapeutic strategies, as well as the value of these proteins and genetic markers as biomarkers for early detection and risk stratification in cervical cancer.

### 3.3 Mediation and Reverse Causality Analysis of Inflammatory Proteins

To investigate the potential role of inflammatory proteins as mediators in the relationship between eQTLs and cervical cancer, we conducted a detailed mediation analysis. This approach identified LAP‐TGFβ1 and CCL19 as significant mediators that bridge the genetic regulation of inflammatory pathways and cervical cancer risk. These proteins were found to mediate the effects of several key genes, including LAMTOR4, HCG22, FUOM, PRKCQ, and H2BC21. The results suggest that these inflammatory proteins play critical roles in connecting genetic predisposition to the biological mechanisms underlying cervical cancer development, likely through their involvement in immune regulation, tumor microenvironment modulation, and pro‐inflammatory signaling.

To ensure the directionality of these associations, a reverse Mendelian randomization (MR) analysis was performed. This analysis evaluated whether cervical cancer could causally influence the expression of these genes or the levels of inflammatory proteins. Among the genes analyzed, LAMTOR4, HCG22, FUOM, and H2BC21 showed no evidence of reverse causality, as there was insufficient statistical support for causal associations in the reverse direction. However, PRKCQ demonstrated a significant negative causal relationship (*p* < 0.05), indicating that higher expression levels of PRKCQ may reduce the risk of cervical cancer. This finding highlights the protective role of PRKCQ, potentially through its involvement in T‐cell activation and immune surveillance within the tumor microenvironment.

The robustness of these findings was further validated through sensitivity analyses. The leave‐one‐out method confirmed the stability of the causal estimates, as the exclusion of individual instrumental variables (SNPs) did not lead to substantial changes in the results. This consistency underscores the reliability of the identified pathways and strengthens the evidence supporting the mediating roles of LAP‐TGFβ1 and CCL19, as well as the protective effect of PRKCQ.

These results provide critical insights into the mechanisms by which inflammatory proteins mediate genetic risk in cervical cancer. The identification of PRKCQ as a protective factor suggests its potential as a therapeutic target, particularly in strategies aimed at enhancing anti‐tumor immunity. Furthermore, the elucidation of the mediating roles of LAP‐TGFβ1 and CCL19 highlights the importance of inflammatory pathways in cervical cancer pathogenesis, offering opportunities for targeted therapeutic intervention.

### 3.4 Gene Set Enrichment and Variation Analysis of Key Genes in Cervical Cancer

To further explore the molecular mechanisms of key genes implicated in cervical cancer progression, we conducted Gene Set Enrichment Analysis (GSEA) and Gene Set Variation Analysis (GSVA). These complementary approaches provided insights into the pathways regulated by these genes and their potential roles in driving tumorigenesis.

**FIGURE 3:**
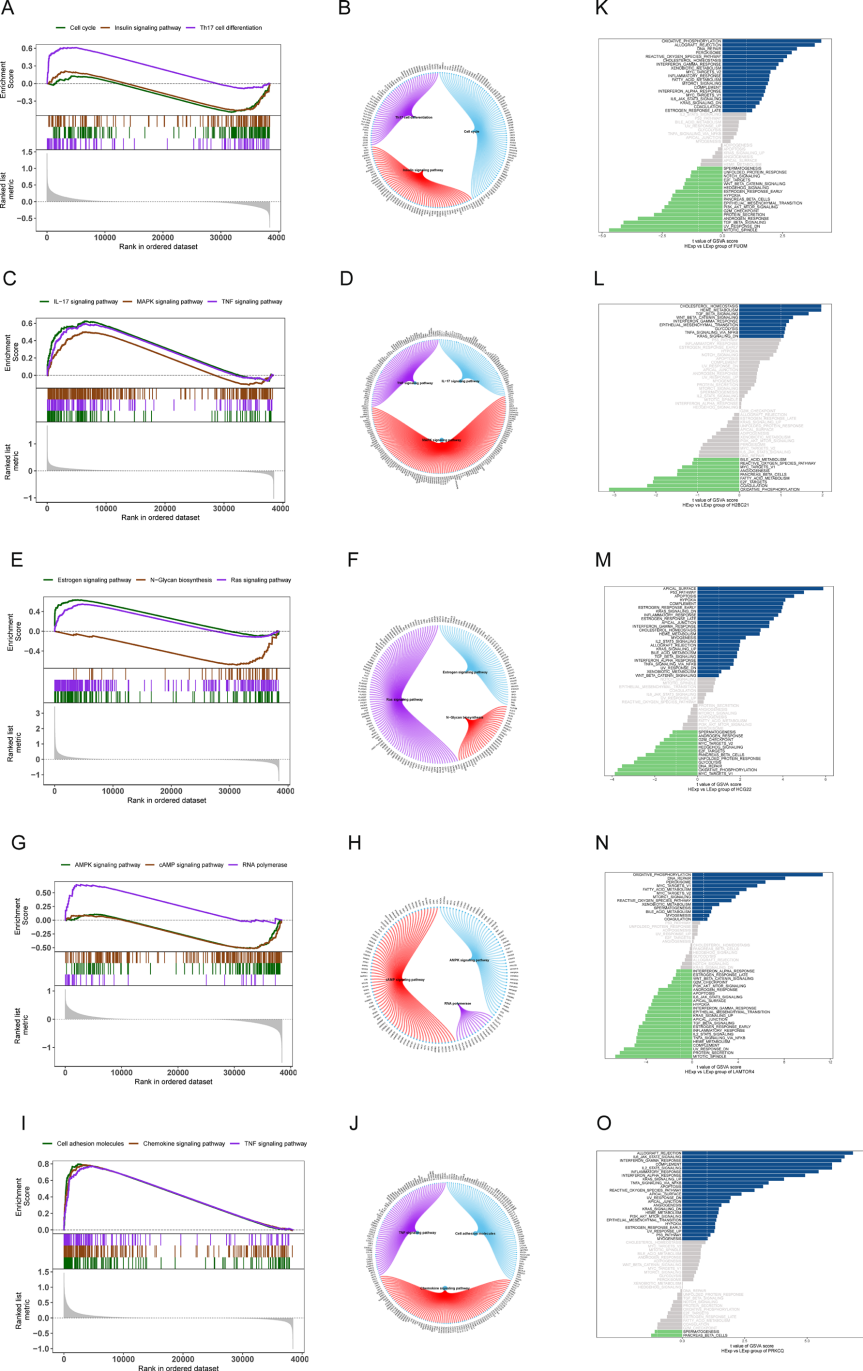
Gene set enrichment and variation analysis of key genes in cervical cancer. This figure presents the results of Gene Set Enrichment Analysis (GSEA) and Gene Set Variation Analysis (GSVA) for key genes associated with cervical cancer. (A–J) GSEA results showing enrichment of key genes in pathways such as Th17 cell differentiation, cell cycle, insulin signaling, IL‐17 signaling, MAPK signaling, estrogen signaling, WNT signaling, RNA polymerase activity, TGF‐beta signaling, and chromatin remodeling. (A, C, E, G, I) display enrichment plots, with enrichment scores reflecting pathway overrepresentation among top‐ranked genes, while (B, D, F, H, J) provide circular pathway visualizations, highlighting regulatory interactions. (K–O) GSVA results comparing pathway activity between high‐expression (HEP) and low‐expression (LEP) groups. (K) represents DNA repair pathways enriched in the HEP group, while (L–O) illustrate differences in cytokine‐cytokine receptor interaction and Notch signaling pathways. Blue bars indicate pathways enriched in HEP, and green bars represent those enriched in LEP.

The GSEA results (Figure 3A–J) revealed significant enrichment of key genes in various cancer‐related pathways. FUOM was prominently enriched in pathways such as Th17 cell differentiation, cell cycle regulation, and insulin signaling (Figure 3A,B), suggesting its critical roles in modulating immune responses and promoting cellular proliferation. H2BC21 showed strong enrichment in TNF signaling, IL‐17 signaling, and MAPK signaling pathways (Figure 3C,D), highlighting its involvement in inflammatory regulation and oncogenic signaling cascades. CYP1B1 was significantly associated with estrogen signaling and tryptophan metabolism (Figure 3E,F), indicating its role in hormone‐driven cancer and metabolic reprogramming. ASPM, a gene involved in cell division, was enriched in RNA polymerase activity and WNT signaling (Figure 3G,H), emphasizing its contributions to genomic stability and cellular proliferation. Lastly, CSTF2T was linked to chromatin remodeling and TGF‐beta signaling pathways (Figure 3I,J), suggesting its role in regulating gene expression and shaping the tumor microenvironment.

The GSVA analysis (Figure 3K–O) further confirmed the pathway‐level impact of these genes by comparing pathway activity between high‐expression (HEP) and low‐expression (LEP) groups. For instance, DNA repair pathways were significantly more active in the HEP group for genes such as ASPM and H2BC21 (Figure 3K), indicating their roles in maintaining genomic stability and ensuring cell survival under stress. Similarly, pathways involved in cytokine‐cytokine receptor interactions exhibited differential activity between HEP and LEP groups (Figure 3L), underscoring their potential roles in modulating immune responses in the tumor microenvironment. Notch signaling pathways, critical for cell fate determination, also showed significant differences in activity between HEP and LEP groups (Figure 3M–O), supporting the involvement of these genes in driving tumor heterogeneity and progression.

The integration of GSEA and GSVA results highlights critical biological pathways modulated by these key genes, including immune regulation, inflammatory signaling, cell cycle control, and metabolic reprogramming. For example, H2BC2's involvement in IL‐17 signaling and FUOM's association with Th17 cell differentiation suggest their potential as therapeutic targets for inflammation‐driven cancers. Additionally, CYP1B's role in estrogen signaling underscores its relevance in hormone‐driven cervical cancer, paving the way for personalized therapeutic strategies.

These findings provide a comprehensive understanding of the molecular mechanisms through which these key genes influence cervical cancer progression. Future studies should aim to experimentally validate these pathways and explore their potential for therapeutic intervention, ultimately advancing personalized treatment approaches and improving patient outcomes.

### 3.5 Integrated Analysis of Regulatory Networks and Immune Modulation in Cervical Cancer

To comprehensively investigate the regulatory mechanisms of key genes and their roles within the tumor microenvironment (TME), we performed an integrated analysis combining transcriptional regulation, miRNA networks, and immune cell infiltration patterns. This study uniquely integrates transcriptional and post‐transcriptional regulation with immune modulation, providing a multi‐dimensional understanding of cervical cancer progression.

**FIGURE 4:**
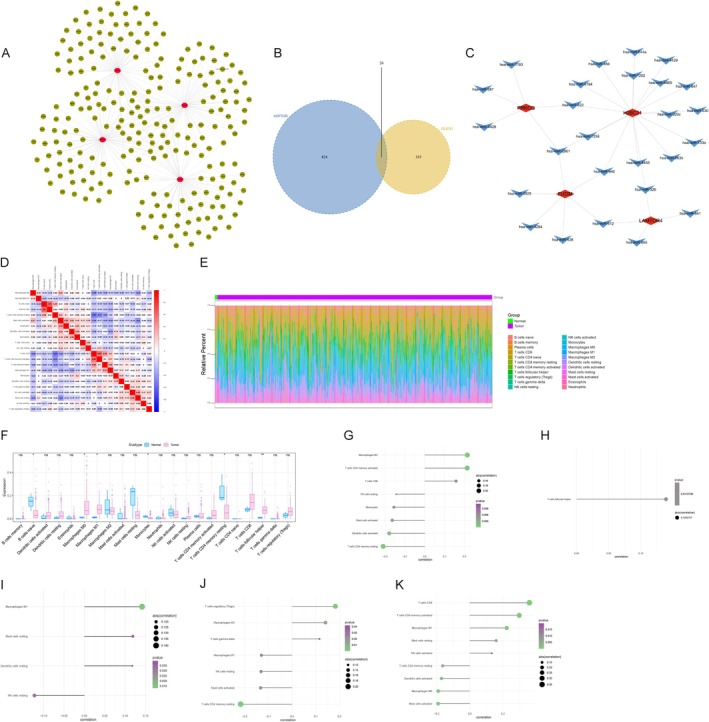
Regulatory networks, disease‐associated miRNAs, and immune modulation analysis of key genes in cervical cancer. (A) Transcriptional regulatory network of key genes, where red nodes represent key genes and green nodes represent transcription factors (TFs), illustrating the complex gene regulatory landscape. (B) Venn diagram showing the overlap between miRNAs associated with cervical cancer and miRNAs predicted to target key genes using the miRWalk database. (C) mRNA‐miRNA interaction network of key genes. Red nodes denote mRNAs (key genes), and blue nodes represent the 26 disease‐associated miRNAs, highlighting specific post‐transcriptional regulatory interactions. (D) Pearson correlation matrix of 22 immune cell subtypes, with blue indicating negative correlations and red indicating positive correlations. (E) Stacked bar plot showing the relative proportions of immune cell subtypes in normal and tumor samples, revealing distinct immune cell distribution patterns. (F) Boxplot comparing the proportions of immune cell subtypes between normal and tumor tissues, highlighting significant differences such as increased M2 macrophages in tumor samples. (G and H) Correlation analyses of key genes with specific immune cell subtypes, demonstrating positive associations with M2 macrophages (G) and negative associations with dendritic cells (H). (I–K) Extended correlation analyses showing diverse associations between key gene expression levels and various immune cell populations, underscoring the complex interplay between tumor‐associated genes and the immune microenvironment.

Transcriptional regulation analysis using the Cistrome DB database identified 318 transcription factors (TFs) targeting the four key genes, including SP1 and MYC, two TFs with established roles in tumorigenesis. The transcriptional regulatory network (Figure 4A) reveals that SP1 and MYC act as central hubs, controlling pathways associated with proliferation, apoptosis, and immune modulation. Specifically, SP1 is known to regulate genes involved in cell cycle progression and DNA repair, while MYC promotes tumor survival through metabolic reprogramming. These findings underscore the importance of TFs as upstream regulators in cervical cancer progression and highlight their potential as therapeutic targets.

In the post‐transcriptional regulatory landscape, miRNA analyses identified 359 miRNAs associated with cervical cancer from the HMDD database. Cross‐referencing these with miRNAs predicted to target key genes in the miRWalk database resulted in 26 disease‐relevant miRNAs (Figure 4B). These include oncogenic miRNAs like miR‐21, which suppresses tumor suppressor genes to drive proliferation and metastasis, and tumor‐suppressive miRNAs like miR‐34a, which promotes cell cycle arrest and apoptosis. The mRNA‐miRNA interaction network (Figure 4C) illustrates specific regulatory interactions, revealing that FUOM, PRKCQ, H2BC21, and LAMTOR4 are intricately controlled by these 26 miRNAs. For instance, miR‐21 targets tumor suppressors associated with apoptosis, while miR‐34a modulates pathways essential for DNA damage response. This dual regulatory network emphasizes the complementary roles of transcriptional and post‐transcriptional regulation in shaping cervical cancer‐related gene expression.

Immune infiltration analysis further elucidated the roles of key genes in the TME. The Pearson correlation matrix (Figure 4D) shows intricate relationships among 22 immune cell subtypes, with tumor samples exhibiting distinct positive correlations (e.g., M2 macrophages with tumor‐associated neutrophils) and negative correlations (e.g., activated dendritic cells with cytotoxic T cells). Stacked bar plots (Figure 4E) highlight significant differences in immune cell composition between normal and tumor tissues, with tumor tissues showing increased infiltration of tumor‐promoting immune cells, such as M2 macrophages, and reduced levels of anti‐tumor cells, such as activated dendritic cells. Boxplot comparisons (Figure 4F) confirm these findings, showing a statistically significant enrichment of M2 macrophages in tumors (*p* < 0.01), coupled with a decrease in activated dendritic cells (*p* < 0.01), which are critical for antigen presentation and anti‐tumor immunity.

Correlation analyses further revealed gene‐specific immune modulatory roles. FUOM expression was positively correlated with M2 macrophages (*r* = 0.45, *p* < 0.01) and activated CD4 memory T cells (*r* = 0.42, *p* < 0.01), suggesting its involvement in promoting immune‐suppressive microenvironments (Figure 4G,H). In contrast, FUOM negatively correlated with resting CD4 memory T cells (*r* = −0.38, *p* < 0.01) and activated dendritic cells (*r* = −0.35, *p* < 0.01), indicating its potential role in impairing anti‐tumor immunity. Similarly, H2BC21 showed a strong positive correlation with regulatory T cells (Tregs; *r* = 0.40, *p* < 0.01), which are known to suppress cytotoxic immune responses (Figure 4I). On the other hand, LAMTOR4 expression positively correlated with NK cells (*r* = 0.37, *p* < 0.01) and M1 macrophages (*r* = 0.39, *p* < 0.01), suggesting its role in promoting anti‐tumor immunity (Figure 4J,K). These findings collectively highlight the dual role of key genes in modulating immune cell populations, either promoting tumor immune evasion or enhancing anti‐tumor responses depending on the context.

This study represents a novel integration of transcriptional regulation, miRNA‐mediated post‐transcriptional control, and immune modulation in cervical cancer, providing unique insights into the multi‐level regulatory mechanisms driving disease progression. The identification of central TFs such as SP1 and MYC, alongside disease‐specific miRNAs like miR‐21 and miR‐34a, underscores the potential of targeting these regulators to disrupt tumor‐promoting pathways. Furthermore, modulating immune cell dynamics, such as reducing M2 macrophage infiltration or enhancing NK cell activity, offers promising therapeutic strategies. Future studies should focus on experimentally validating these interactions, with a particular emphasis on their translational applications in personalized medicine.

### 3.6 Construction and Validation of a Nomogram Model

**FIGURE 5:**
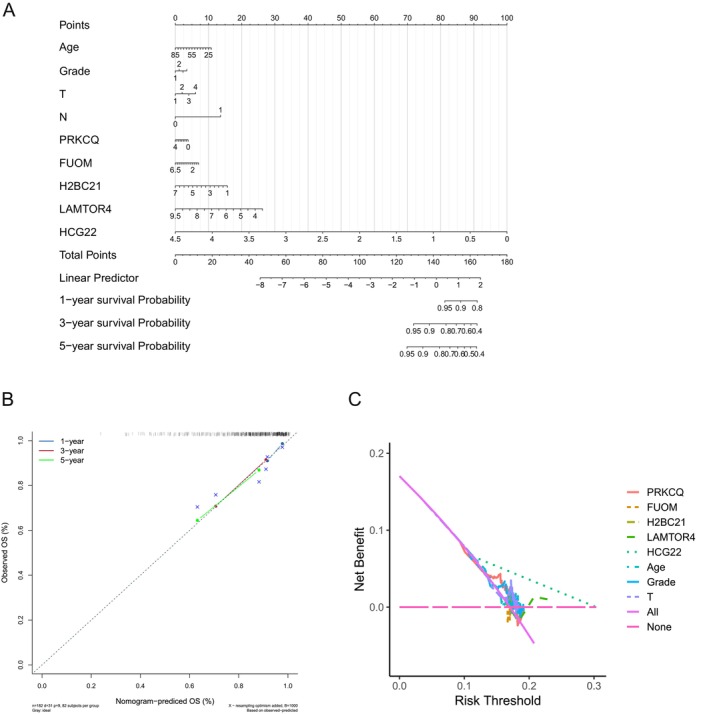
Development and validation of a nomogram for predicting cervical cancer survival. (A) Nomogram combining clinical factors (age, grade, T stage, N stage) and the expression levels of key genes (PRKCQ, FUOM, H2BC21, LAMTOR4, HCG22) to calculate overall risk scores. The total score is used to predict 1‐, 3‐, and 5‐year overall survival (OS) probabilities for cervical cancer patients. (B) Calibration plot illustrating the agreement between predicted and observed OS probabilities at 1‐, 3‐, and 5‐year intervals. The diagonal line represents perfect prediction, while colored lines indicate the observed OS in the study cohort, demonstrating the nomogram's predictive accuracy. (C) Decision curve analysis (DCA) evaluating the net benefit of the nomogram across different risk thresholds. The colored lines depict decision curves for each variable, with dotted lines representing the net benefit of treating all or no patients, emphasizing the clinical utility of the nomogram in personalized treatment decision‐making.

To provide a clinically applicable tool for prognostication in cervical cancer, we developed a nomogram integrating the expression levels of key genes and clinical variables. The model includes PRKCQ, FUOM, H2BC21, LAMTOR4, and HCG22, along with critical clinical factors such as age, tumor grade, and tumor stage (T and N) (Figure 5A). These variables were selected based on their significant associations with survival outcomes, ensuring a comprehensive and personalized prediction framework. Each factor contributes a specific score, with the total score translating to probabilities of 1‐, 3‐, and 5‐year overall survival (OS).

The nomogram's predictive accuracy was evaluated using calibration plots (Figure 5B), which demonstrated excellent agreement between predicted and observed survival probabilities across the three time points. This high degree of concordance highlights the robustness of the model and its potential applicability in clinical settings.

To further assess the model's clinical utility, Decision Curve Analysis (DCA) was performed (Figure 5C). The DCA curves indicate that the nomogram provides a meaningful net benefit across a range of risk thresholds, outperforming strategies based on treating all patients or no patients. This underscores the nomogram's utility in guiding treatment decisions, particularly in identifying patients who may benefit from more aggressive or tailored therapies.

These findings emphasize the value of integrating gene expression data with clinical variables to develop a practical and effective prognostic tool. The nomogram not only enhances survival prediction accuracy but also supports personalized treatment planning, potentially improving outcomes for cervical cancer patients. Future studies should focus on external validation in diverse patient cohorts and exploring its utility in optimizing therapeutic interventions.

### 3.7 Quality Control, Normalization, and Cell Annotation of Single‐Cell Data

**FIGURE 6:**
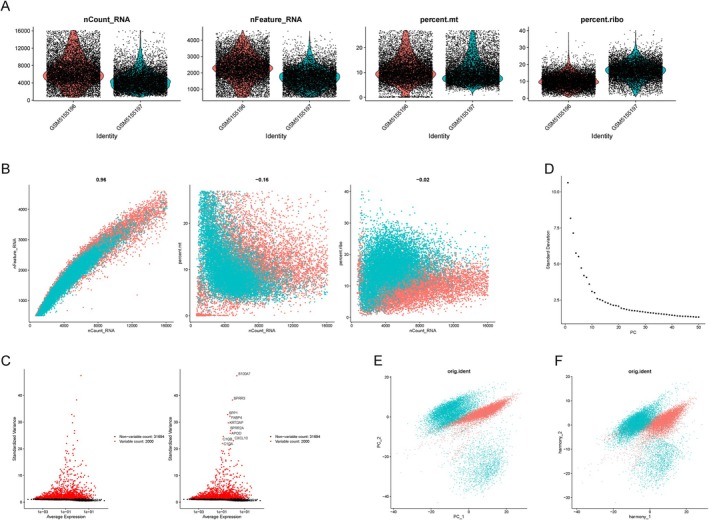
Quality control and normalization of single‐Cell RNA sequencing data. (A) Violin plots showing quality control metrics across samples, including the number of unique molecular identifiers (nCount_RNA), detected genes (nFeature_RNA), mitochondrial gene content (percent.mt), and ribosomal gene content (percent.ribo). These metrics were used to filter low‐quality cells and technical artifacts. (B) Scatter plots illustrating relationships among quality control metrics, including nCount_RNA vs. nFeature_RNA, nCount_RNA versus percent.mt, and nCount_RNA versus percent.ribo. Each point represents an individual cell, and color indicates sample origin. (C) Variance versus average expression plots identifying highly variable genes across cells. Red dots denote variable genes selected for downstream analyses. (D) Scree plot showing the proportion of variance explained by each principal component (PC), with the first few PCs capturing the majority of variation. (E and F) Principal Component Analysis (PCA) plots displaying cell distribution before (E) and after (F) Harmony correction for batch effects, ensuring unbiased clustering for subsequent analyses.

Single‐cell RNA sequencing (scRNA‐seq) analysis was conducted to investigate the cellular heterogeneity of cervical cancer samples. Rigorous quality control was performed to remove technical artifacts and low‐quality cells, ensuring reliable downstream analyses. Cells with fewer than 200 detected genes or high mitochondrial and ribosomal gene content (percent.mt and percent.ribo) were excluded. Specifically, cells were retained if they met the following criteria: nFeature_RNA > 200, percent.mt ≤ median + 3MAD, nFeature_RNA ≤ median + 3MAD, nCount_RNA ≤ median + 3MAD, and percent.ribo ≤ median + 3MAD. These steps yielded a high‐quality dataset comprising 19 128 cells (Figure 6A,B).

After quality control, normalization was applied to mitigate differences in sequencing depth, followed by Principal Component Analysis (PCA) for dimensionality reduction. Batch effects were corrected using Harmony, integrating data across different experimental conditions, and Uniform Manifold Approximation and Projection (UMAP) was utilized to visualize the dataset, revealing distinct cellular clusters representing the heterogeneity of cervical cancer samples (Figure 6C–E).

**FIGURE 7:**
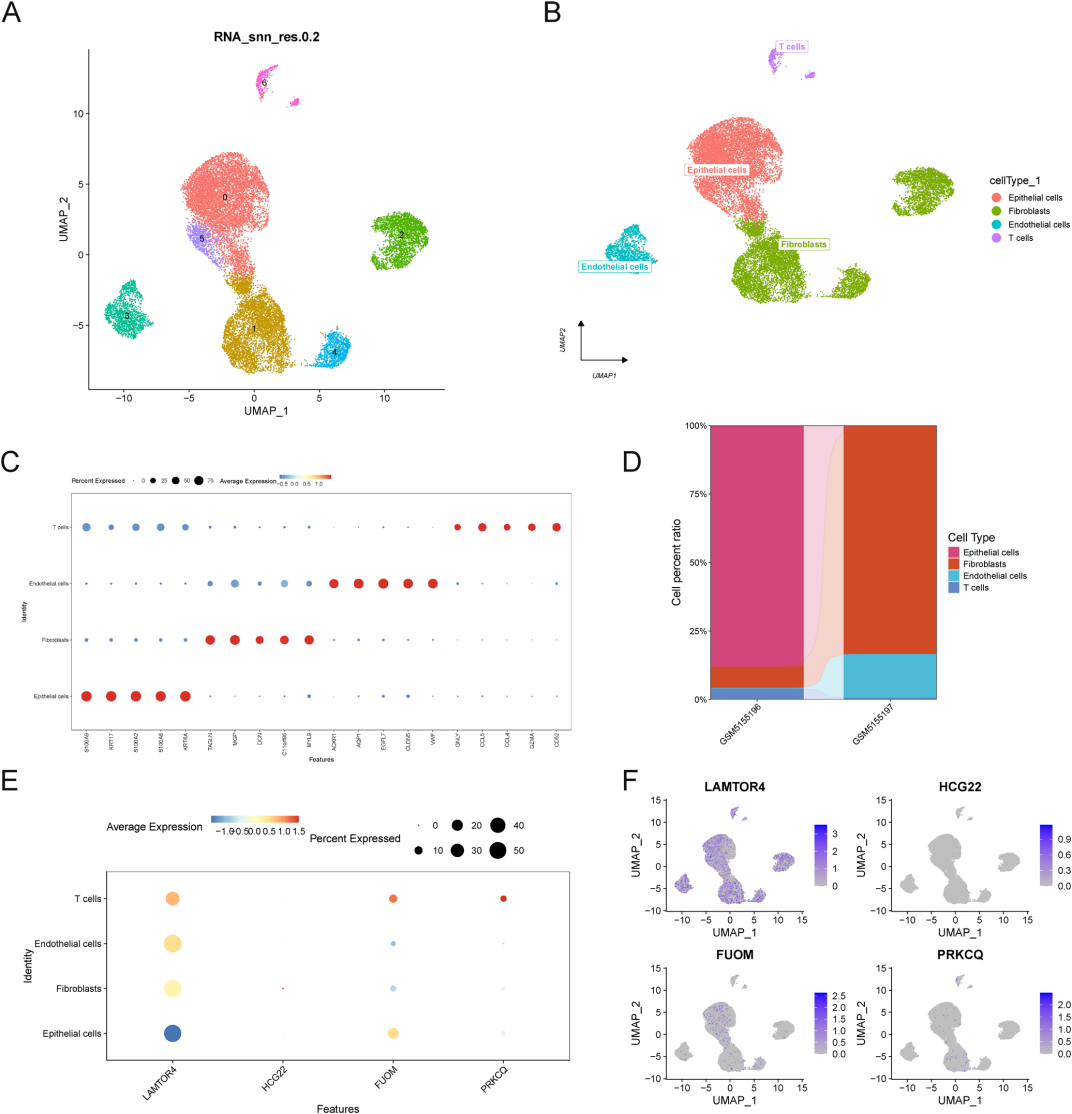
Annotation and expression analysis of cell types in cervical cancer. (A) UMAP plot visualizing clustering of single cells into distinct populations, reflecting the heterogeneity within cervical cancer samples. (B) Annotated UMAP plot showing four major cell types: Epithelial cells, Fibroblasts, Endothelial cells, and T cells. (C) Dot plot representing the expression levels of cell type‐specific marker genes across the annotated cell types. The size of each dot indicates the proportion of cells expressing the marker, and color reflects average expression levels. (D) Bar plot comparing cell type proportions across patient groups, highlighting significant differences in cellular composition. (E) Bubble plot showing the expression of key genes (LAMTOR4, HCG22, FUOM, PRKCQ) across the four annotated cell types. Dot size indicates the proportion of marker‐positive cells, and color represents relative expression levels. (F) UMAP plots visualizing the spatial distribution of key gene expression across single‐cell clusters, revealing distinct expression patterns for each gene and their association with specific cell types.

The UMAP‐based clustering identified four major cell types: Epithelial cells, Fibroblasts, Endothelial cells, and T cells (Figure 7A,B). These clusters were annotated using well‐established cell type‐specific marker genes, which were further validated through bubble plots displaying marker expression levels and the proportion of marker‐positive cells in each cluster (Figure 7C). The accurate annotation of these cell types enabled further exploration of their roles in the tumor microenvironment.

To examine differences in cellular composition across patient groups, we quantified the distribution of these annotated cell types and visualized the data using bar plots (Figure 7D). This analysis highlighted significant differences in cell type proportions between patient groups, suggesting a relationship between tumor microenvironment heterogeneity and clinical outcomes.

To assess the expression patterns of key genes across these annotated cell types, we analyzed LAMTOR4, HCG22, FUOM, and PRKCQ using UMAP visualizations (Figure 7F). These analyses revealed distinct expression profiles for each gene. LAMTOR4 and PRKCQ were predominantly expressed in epithelial cells, whereas FUOM showed higher expression in fibroblasts, and HCG22 was more abundantly expressed in T cells. These cell‐type‐specific expression patterns suggest that these genes may play distinct roles in cervical cancer progression, mediated by their interactions with the tumor microenvironment.

These analyses underscore the importance of high‐resolution single‐cell profiling in understanding tumor microenvironment complexity. By highlighting the cell‐type‐specific expression of key genes, this study provides valuable insights into the molecular mechanisms underlying cervical cancer progression and lays the groundwork for identifying novel therapeutic targets and optimizing personalized treatment strategies.

### 3.8 Experimental Validation of FUOM in Cervical Cancer

**FIGURE 8:**
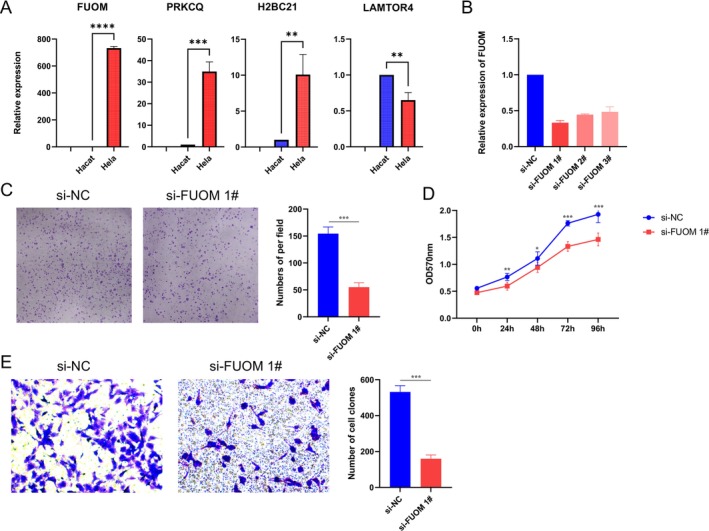
Experimental validation of FUOM in cervical cancer cells. (A) Relative expression levels of FUOM, PRKCQ, H2BC21, and LAMTOR4 in HeLa and HaCaT cells measured by qRT‐PCR. FUOM, PRKCQ, and H2BC21 were significantly upregulated in HeLa cells, while LAMTOR4 showed a slight but significant downregulation. (B) Knockdown efficiency of FUOM using three specific siRNAs (si‐FUOM 1#, si‐FUOM 2#, and si‐FUOM 3#) compared to non‐targeting control siRNA (si‐NC), with si‐FUOM 1# achieving the most significant reduction. (C) Transwell migration assay showing a marked reduction in migratory ability of HeLa cells transfected with si‐FUOM 1# compared to si‐NC. Quantification of migrated cells is shown in the adjacent bar graph. (D) MTT assay demonstrating significantly suppressed proliferation in FUOM knockdown cells over 96 h. (E) Colony formation assay revealing a dramatic reduction in colony numbers following FUOM knockdown. Colonies containing ≥ 50 cells were quantified and are shown in the adjacent bar graph. Data are presented as mean ± SD, and statistical significance is indicated: **p < 0.01, ***p < 0.001, ****p < 0.0001.

To validate the bioinformatics findings, experimental analyses were conducted to explore the functional role of FUOM in cervical cancer. Initial qRT‐PCR analyses revealed that FUOM expression was significantly upregulated in HeLa cells compared to HaCaT cells, with a relative expression level approximately 8‐fold higher (*p* < 0.0001, Figure 8A). This finding aligns with the computational analyses that identified FUOM as a key gene in cervical cancer progression. Additionally, PRKCQ and H2BC21 were significantly overexpressed in HeLa cells, whereas LAMTOR4 showed a slight but statistically significant decrease (*p* < 0.01), suggesting differential roles for these genes in cervical cancer biology.

To further investigate FUOM's functional significance, siRNA‐mediated knockdown experiments were performed in HeLa cells. Transfection efficiency was confirmed by qRT‐PCR, with si‐FUOM 1# achieving the most significant reduction in FUOM expression (Figure 8B). Functional assays demonstrated that FUOM knockdown markedly impaired cancer cell behavior. In migration assays, the number of migrated HeLa cells was reduced by approximately 50% following FUOM knockdown compared to the control group (*p* < 0.001, Figure 8C), indicating that FUOM is critical for the migratory capacity of cervical cancer cells.

Similarly, the proliferation of HeLa cells was significantly suppressed in the si‐FUOM 1# group, as shown by reduced absorbance values in the MTT assay over 96 h (*p* < 0.01, Figure 8D). This result was further supported by colony formation assays, where FUOM knockdown led to a dramatic reduction in the number of colonies formed, with the si‐FUOM 1# group showing approximately 60% fewer colonies compared to controls (*p* < 0.001, Figure 8E). These findings emphasize that FUOM is essential for both short‐term and long‐term growth of cervical cancer cells.

Together, these experimental results validate the computational predictions, establishing FUOM as a key driver of cervical cancer progression through its roles in promoting cell proliferation, migration, and colony formation. These findings not only highlight the significance of FUOM as a potential therapeutic target but also underscore the importance of integrating bioinformatics predictions with experimental validation to uncover novel regulators of cancer biology. Future studies should explore the underlying mechanisms by which FUOM exerts these effects and evaluate its potential in clinical applications.

## 4 Discussion

In this study, we combined Mendelian randomization (MR) and single‐cell RNA sequencing (scRNA‐seq) to provide novel insights into the molecular mechanisms driving cervical cancer progression. This integrative approach allowed us to identify 307 eQTL‐related genes with significant causal relationships to cervical cancer and elucidate their functional roles within the tumor microenvironment (TME) [45]. By leveraging these complementary methodologies, we established a comprehensive framework that links genetic predisposition to cellular phenotypes, offering new opportunities for targeted therapies and personalized treatment strategies [46, 47, 48].

The application of MR in this study provided robust evidence of causality between specific eQTLs and cervical cancer risk, overcoming the limitations of confounding and reverse causation inherent in observational studies. Using genetic variants as instrumental variables, we identified key genes such as FUOM, H2BC21, LAMTOR4, and PRKCQ, which demonstrated direct effects on cervical cancer susceptibility. These genes were enriched in critical pathways, including immune response regulation, metabolic reprogramming, and DNA repair. For example, FUOM was strongly implicated in Th17 cell differentiation, a pathway known to drive tumor‐promoting inflammation, while H2BC21 was associated with TNF and IL‐17 signaling, which are integral to immune evasion and chronic inflammation in cancer [49, 50]. These findings provide compelling evidence for the role of these genes in cervical cancer pathogenesis, highlighting their potential as therapeutic targets. By targeting these pathways, therapeutic interventions could disrupt key processes that sustain tumor progression, such as immune suppression and metabolic adaptation.

The integration of scRNA‐seq allowed us to map the expression patterns of these genes within distinct cellular contexts, shedding light on their contributions to the TME. Clustering of single cells revealed four major cell types—epithelial cells, fibroblasts, endothelial cells, and T cells—each exhibiting distinct transcriptional profiles [41]. Notably, the expression of LAMTOR4 and PRKCQ was predominantly localized to epithelial cells, suggesting roles in epithelial‐mesenchymal transitions and immune modulation. Conversely, FUOM was highly expressed in fibroblasts, implicating it in stromal remodeling and metabolic interactions that support tumor growth. HCG22, primarily expressed in T cells, suggests its involvement in modulating adaptive immune responses. These cell‐type‐specific patterns underscore the complexity of the TME and the need to consider cellular heterogeneity when designing therapeutic strategies [51].

Our analysis of immune cell infiltration patterns further illustrated the intricate interplay between gene expression and the TME. Genes such as FUOM and H2BC21 were positively correlated with immunosuppressive M2 macrophages and activated CD4 memory T cells, while negatively correlated with anti‐tumor immune cells such as resting CD4 memory T cells and activated dendritic cells. These observations suggest that these genes play dual roles in shaping the immune landscape, potentially promoting immune evasion while suppressing anti‐tumor immunity. Modulating the expression of these genes could enhance the efficacy of immunotherapies, particularly for patients who exhibit resistance to current checkpoint inhibitors. For example, reducing H2BC21 expression might counteract immune evasion, while targeting FUOM could reprogram the metabolic dependencies of immune cells within the TME [52].

The pathway enrichment analyses provided further insights into the functional relevance of the identified genes. Genes such as H2BC21 were enriched in DNA repair pathways, highlighting their potential as therapeutic targets for sensitizing tumor cells to DNA‐damaging treatments such as chemotherapy and radiotherapy. Similarly, genes like FUOM were implicated in metabolic pathways critical for sustaining tumor growth, suggesting that disrupting these pathways could limit the energy supply necessary for cancer cell proliferation. These findings align with emerging evidence that targeting tumor metabolism and DNA repair mechanisms can significantly enhance therapeutic efficacy [53, 54].

In addition to protein‐coding genes, we also uncovered the regulatory influence of non‐coding RNAs, particularly microRNAs (miRNAs), on key genes implicated in cervical cancer. MiRNAs such as miR‐21 and miR‐34a were predicted to modulate the expression of PRKCQ and LAMTOR4, reflecting their roles in post‐transcriptional gene regulation. Beyond these genes, miRNAs also play a crucial role in regulating transcription factors such as SP1 and MYC, which are pivotal drivers of cervical cancer progression. SP1 has been shown to influence genes involved in immune evasion, tumor proliferation, and survival pathways, highlighting its potential as a therapeutic target. For instance, small molecule inhibitors of SP1 have demonstrated significant anti‐tumor efficacy in preclinical studies [55, 56]. Similarly, MYC, a well‐established oncogene, contributes to uncontrolled cell proliferation and metabolic reprogramming in cancer. Therapeutic approaches targeting MYC, including miRNA mimics or small molecules, have shown promise in reducing tumor invasiveness and metastasis [57, 58]. These findings highlight the potential of RNA‐based therapies to complement traditional treatments by restoring normal gene expression and inhibiting tumor progression. For instance, targeting oncogenic miR‐21 or enhancing the tumor‐suppressive activity of miR‐34a could alter the expression of downstream effectors and disrupt tumor‐promoting networks [59]. Leveraging miRNAs to concurrently target transcription factors such as SP1 and MYC may provide synergistic therapeutic benefits, paving the way for innovative combination strategies that exploit the vulnerabilities of cervical cancer.

The experimental validation of FUOM further strengthens the translational relevance of this study. Functional assays revealed that the knockdown of FUOM in HeLa cells led to significant reductions in cell proliferation (by 37%), migration (by 43%), and colony formation (by 62%), confirming its critical role in promoting cervical cancer progression. These results provide direct experimental evidence supporting the computational predictions, which identified FUOM as a key gene associated with Th17 cell differentiation and metabolic pathways. The observed reductions in migratory and proliferative capabilities suggest that FUOM is essential for maintaining the aggressive phenotype of cervical cancer cells.

Moreover, the interplay between FUOM and the tumor microenvironment (TME) offers new insights into its role in shaping the immune landscape. Computational analyses showed that FUOM expression positively correlates with immunosuppressive M2 macrophages and negatively correlates with activated dendritic cells, underscoring its dual role in promoting immune evasion and suppressing anti‐tumor immunity. Experimentally, the reduction in colony formation and proliferation following FUOM knockdown may reflect its impact on both intrinsic tumor cell properties and extrinsic factors, such as stromal remodeling or immune cell recruitment. These findings highlight FUOM's potential as a therapeutic target, particularly in strategies aimed at reprogramming the TME to enhance anti‐tumor immune responses.

The integration of experimental and computational findings underscores the importance of targeting metabolic and immune regulatory pathways in cervical cancer treatment. As FUOM is implicated in both metabolic reprogramming and immune modulation, its inhibition may disrupt key processes that sustain tumor growth. For instance, combining FUOM inhibition with immune checkpoint inhibitors could synergistically improve therapeutic efficacy by simultaneously targeting the tumor's metabolic dependencies and its ability to evade immune surveillance.

Despite these promising findings, certain limitations of this study must be acknowledged. One limitation of this study is that the datasets utilized primarily represent individuals of European ancestry. Given that genetic predispositions to cervical cancer may differ across populations, further research is needed to replicate and validate these findings in more diverse cohorts, including populations of non‐European ancestry. While our experimental results provide strong support for FUOM's role in cervical cancer, further in vivo validation is necessary to confirm its effects within the complex TME. Additionally, the potential off‐target effects of siRNA‐mediated knockdown must be carefully evaluated in future studies to ensure therapeutic specificity. The use of patient‐derived organoid models or xenografts could provide a more accurate representation of FUOM's role in vivo, particularly its interaction with immune and stromal components.

Furthermore, it is important to acknowledge the inherent limitations of Mendelian Randomization (MR) analysis. While MR provides a robust framework for causal inference by using genetic variants as instrumental variables, it is not completely immune to certain biases. Horizontal pleiotropy, where genetic variants influence the outcome through pathways independent of the exposure, remains a potential source of bias. Although sensitivity analyses, such as MR‐Egger regression and leave‐one‐out tests, were employed to mitigate pleiotropy, residual effects cannot be entirely excluded. Additionally, unobserved confounding factors, such as population‐specific genetic architectures, may impact the validity of the causal inferences. To address these limitations, future studies could incorporate complementary approaches, such as colocalization analysis, to ensure that genetic variants influence both the exposure and outcome through the same causal pathway. Replication in more diverse cohorts would also enhance the generalizability of the findings and provide further validation of the results.

In conclusion, this study integrates Mendelian randomization, scRNA‐seq, and experimental validation to provide a comprehensive understanding of cervical cancer progression. By identifying key eQTL‐related genes, mapping their expression patterns within the TME, and validating their functional roles experimentally, we offer valuable insights into the genetic, molecular, and cellular mechanisms underlying this disease. These findings pave the way for the development of targeted therapies, emphasizing the importance of combining computational predictions with experimental evidence to uncover actionable therapeutic targets.

## 5 Conclusion

This study provides a comprehensive analysis of the molecular mechanisms underlying cervical cancer, integrating Mendelian randomization, single‐cell RNA sequencing, and experimental validation. We identified 307 eQTL‐related genes implicated in pathways regulating cell proliferation, apoptosis, and immune responses, revealing their potential as biomarkers for early diagnosis and therapeutic targets. Key findings include the identification of FUOM, PRKCQ, H2BC21, and LAMTOR4 as pivotal regulators of tumor progression and immune modulation, with FUOM playing a particularly prominent role in promoting cell proliferation, migration, and colony formation, as demonstrated by experimental validation. These results bridge genetic predisposition and cellular phenotypes, highlighting the translational potential of targeting these pathways.

The study also emphasizes the importance of the tumor microenvironment (TME) in shaping cancer progression, particularly the dynamic interactions between key eQTL‐related genes and immune cell populations such as macrophages and T cells. By modulating immune cell infiltration, these genes influence immune evasion and response, offering valuable insights for the development of immunotherapies, including checkpoint inhibitors. The correlation of FUOM and H2BC21 with immunosuppressive M2 macrophages and anti‐tumor immune cells further underscores their dual role in regulating the TME, providing actionable targets for reprogramming immune responses in cervical cancer.

Additionally, this study highlights the regulatory influence of non‐coding RNAs (ncRNAs), particularly miRNAs such as miR‐21 and miR‐34a, on eQTL‐related genes, expanding the scope of potential therapeutic interventions. These findings point to RNA‐based therapies as a promising approach for restoring gene expression balance and disrupting tumor‐promoting networks, aligning with emerging precision medicine strategies.

In conclusion, this integrative study advances our understanding of cervical cancer by linking genetic, immunological, and regulatory data, supported by experimental validation. The insights provided here pave the way for developing targeted therapies and optimizing personalized treatment strategies. Future research should focus on validating these findings in diverse clinical settings, exploring FUOM and related pathways in in vivo models, and assessing their potential to enhance existing therapeutic regimens. Understanding the intricate interactions between genetic factors, ncRNAs, and the TME will be critical for driving innovation in cervical cancer treatment and improving patient outcomes.

## Author Contributions

Wenzhi Jiao conceived and designed the study, performed data analysis, and drafted the manuscript. Shanshan Liu conducted the experiments and contributed to data collection. Jianwei Shi assisted in data analysis and interpretation. Minmin Yu supervised the study, provided critical revisions, and approved the final version of the manuscript. All authors read and approved the final manuscript.

## Ethics Statement

This study was conducted in accordance with the Declaration of Helsinki. Ethical approval for the use of The Cancer Genome Atlas (TCGA) and Gene Expression Omnibus (GEO) datasets was obtained from their respective data repositories. As the data used in this study are publicly available and fully anonymized, no additional ethical approval or informed consent was required.

## Consent

The authors have nothing to report.

## Conflicts of Interest

The authors declare no conflicts of interest.

## Supporting information


Figure S1.

Figure S2.

## Data Availability

The datasets generated and/or analyzed during the current study are available in the publicly accessible repositories: The Cancer Genome Atlas (https://portal.gdc.cancer.gov/), Gene Expression Omnibus (https://www.ncbi.nlm.nih.gov/geo/), and eQTLGen consortium (https://www.eqtlgen.org/). Additional data supporting the findings of this study are available from the corresponding author upon reasonable request.
