# Supplementary figures and images for: Integrative Genomic and Functional Approaches Identify FUOM as a Key Driver and Therapeutic Target in Cervical Cancer
Source: Cancer Rep (Hoboken). 2025 Aug 16;8(8):e70306. doi: 10.1002/cnr2.70306 (PMC12357170; doi:10.1002/cnr2.70306)

A

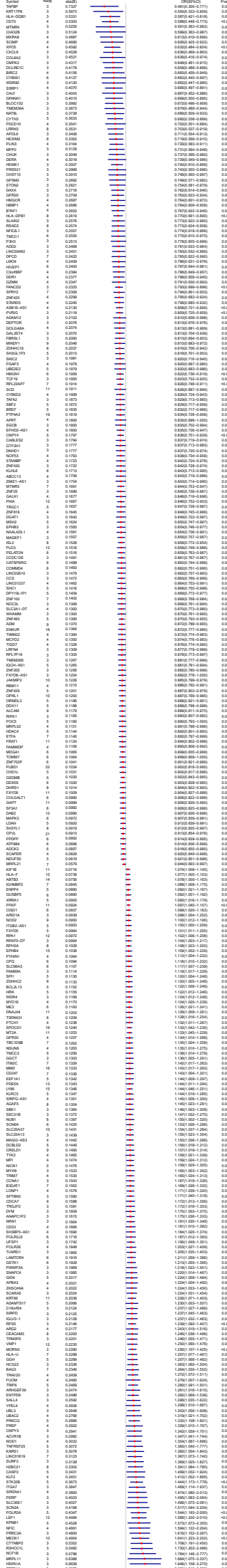

Supplement: Supplementary file 1 — Figure S1. [file CNR2-8-e70306-s002.pdf]

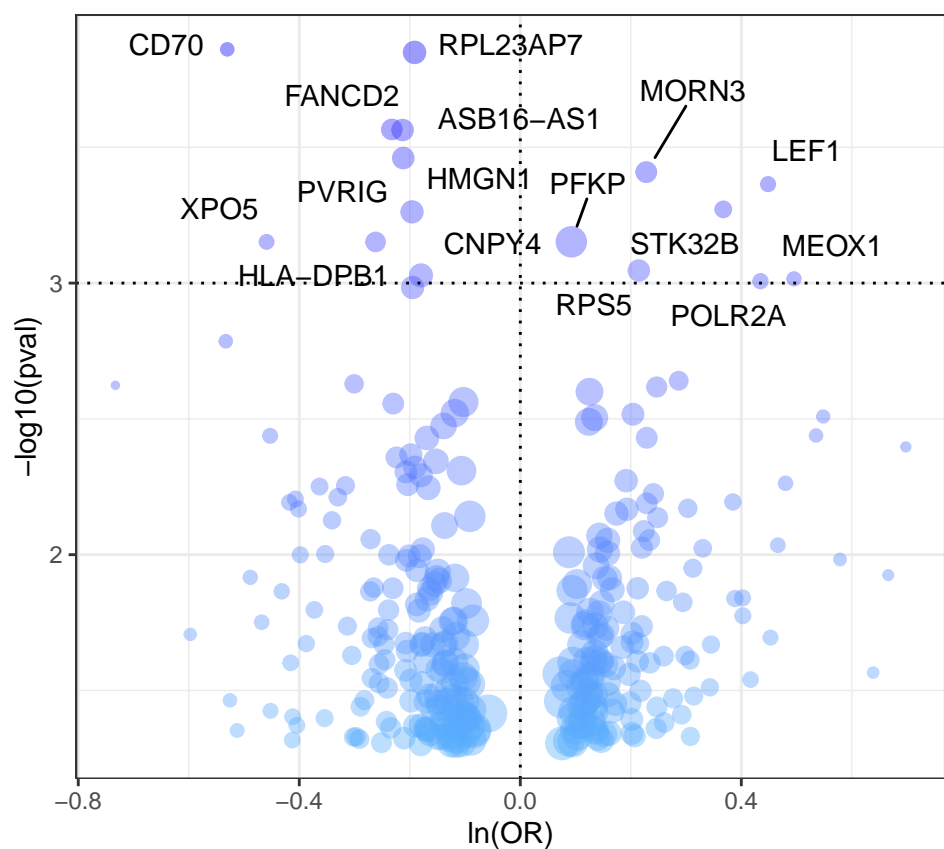

Supplement: Supplementary file 2 — Figure S2. [file CNR2-8-e70306-s001.pdf]
